# COVID-19 Vaccine Hesitancy in Canada: Content Analysis of Tweets Using the Theoretical Domains Framework

**DOI:** 10.2196/26874

**Published:** 2021-04-13

**Authors:** Janessa Griffith, Husayn Marani, Helen Monkman

**Affiliations:** 1 Women's College Hospital Institute for Health Systems Solutions and Virtual Care Women’s College Hospital Toronto, ON Canada; 2 Institute of Medical Science Faculty of Medicine University of Toronto Toronto, ON Canada; 3 Health Information Management Faculty of Health Sciences Douglas College Vancouver, BC Canada; 4 Institute of Health Policy, Management and Evaluation Dalla Lana School of Public Health University of Toronto Toronto, ON Canada; 5 School of Health Information Science Faculty of Human and Social Development University of Victoria Victoria, BC Canada

**Keywords:** vaccine hesitancy, vaccine, COVID-19, immunization, Twitter, infodemiology, infoveillance, social media, behavioral science, behavior, Canada, content analysis, framework, hesitancy

## Abstract

**Background:**

With the approval of two COVID-19 vaccines in Canada, many people feel a sense of relief, as hope is on the horizon. However, only about 75% of people in Canada plan to receive one of the vaccines.

**Objective:**

The purpose of this study is to determine the reasons why people in Canada feel hesitant toward receiving a COVID-19 vaccine.

**Methods:**

We screened 3915 tweets from public Twitter profiles in Canada by using the search words “vaccine” and “COVID.” The tweets that met the inclusion criteria (ie, those about COVID-19 vaccine hesitancy) were coded via content analysis. Codes were then organized into themes and interpreted by using the Theoretical Domains Framework.

**Results:**

Overall, 605 tweets were identified as those about COVID-19 vaccine hesitancy. Vaccine hesitancy stemmed from the following themes: concerns over safety, suspicion about political or economic forces driving the COVID-19 pandemic or vaccine development, a lack of knowledge about the vaccine, antivaccine or confusing messages from authority figures, and a lack of legal liability from vaccine companies. This study also examined mistrust toward the medical industry not due to hesitancy, but due to the legacy of communities marginalized by health care institutions. These themes were categorized into the following five Theoretical Domains Framework constructs: knowledge, beliefs about consequences, environmental context and resources, social influence, and emotion.

**Conclusions:**

With the World Health Organization stating that one of the worst threats to global health is vaccine hesitancy, it is important to have a comprehensive understanding of the reasons behind this reluctance. By using a behavioral science framework, this study adds to the emerging knowledge about vaccine hesitancy in relation to COVID-19 vaccines by analyzing public discourse in tweets in real time. Health care leaders and clinicians may use this knowledge to develop public health interventions that are responsive to the concerns of people who are hesitant to receive vaccines.

## Introduction

The approval of the Pfizer-BioNTech and Moderna vaccines sent waves of excitement and relief across the world. However, some people remain hesitant about receiving a vaccine for COVID-19 [1,2]. The World Health Organization noted in 2019 that one of the greatest threats to global health was vaccine hesitancy [3]. Emerging international evidence on COVID-19 vaccine hesitancy suggests that there is a range of reasons for this reluctance, including doubts about the safety and efficacy of the vaccine, political or pharmaceutical mistrust, belief in natural immunity, and the belief that the virus is mild or not life-threatening [4-6].

For herd immunity to any communicable disease to be effective, a considerable portion of the population needs to be vaccinated or have antibodies present from being recently infected. Achieving herd immunity is difficult when a large portion of the public is not vaccinated. For herd immunity to be effective for measles and polio, 95% and 80% of the population need to be vaccinated, respectively [7]. The exact percentage required for herd immunity to COVID-19 is difficult to estimate [7].

A Statistics Canada survey conducted in September 2020 (before a vaccine was approved) indicated that 75% of Canadians were either likely or somewhat likely to receive a vaccination [8]. An Angus Reid Institute [4] study conducted between December 8 and 11, 2020 found that 48% of Canadians sampled wanted to be vaccinated immediately if a vaccine was available, and 31% wanted to be vaccinated but preferred to wait. Additionally, 7% of respondents indicated that they were unsure if they would receive a vaccination, and 14% indicated that they would not get vaccinated [4].

In the context of influenza vaccinations, there remains a broad, ethical imperative to respect others’ agency over personal health decisions (eg, choosing to not get vaccinated). However, from a public health ethics perspective, the decision to not be vaccinated creates a conflict between population safety and personal liberty [9]. As of yet, COVID-19 vaccination has not been deemed mandatory by any nation, but conversations about whether such a public mandate should exist are emerging [10]. Whether vaccines are mandated, it is worthwhile for public institutions to understand how to change behaviors concerning vaccine hesitancy to ensure that informed decision-making practices are being exercised.

Previous research has suggested that behavioral change interventions are more successful when they are grounded in theory [11]. Thus, we selected a behavioral change framework to guide this study. The Theoretical Domains Framework (TDF) was selected because of its ability to help identify the barriers and facilitators to behavior change while taking into account social and environmental factors [12]. Other public health interventions have used the TDF. For example, Garbutt et al [13] used this framework to improve human papillomavirus vaccine uptake in primary care settings. The use of such theories can facilitate the development of comprehensive health education programs [11], but this requires correctly identifying the attributes of individuals and their surroundings, which influence behavioral patterns [14]. As Bandura [15] and other behavioral theorists have posited, social norms, social relationships, and social networks have a substantial and persistent influence on behaviors [15]. It is worth understanding public discourse about vaccine hesitancy in order to develop interventions that are responsive to the needs of the population and effectively address their concerns.

In the past decade, there has been a particular interest in the utility of Twitter as a tool for monitoring and surveilling public health [16], detecting trends [17], conducting research, and disseminating information [18,19]. A systematic review of using Twitter data for health research found that most studies were in the overlapping fields of public health (23%) and infectious disease (20%) [18]. With 187 million active users worldwide as of January 2021 [20], Twitter has become a powerful social network for disseminating important public health information.

Since the start of the COVID-19 pandemic, social networking platforms like Facebook and YouTube have become stricter with their oversight of the spread of COVID-19 misinformation by deleting false information and providing hyperlinks to government websites containing credible and validated information on COVID-19. Twitter took a similar screening approach in May 2020 [21], yet the scale, spread, and speed of information sharing has made this process challenging. Further, at the start of the pandemic, Twitter introduced a system for verifying COVID-19 experts (indicated with a blue checkmark), including physicians, epidemiologists, scientists, and academics, to provide credible information concerning COVID-19 [22]. Yet, there continues to be influential individuals who have also been verified by Twitter and have enough public credibility to contradict expert opinions or present false information.

We can combat the spread of misinformation by creating targeted approaches to changing behaviors and promoting the understanding of vaccines. Thus, the purpose of this study was to identify the reasons behind vaccine hesitancy among people in Canada by conducting a content analysis of tweets through the lens of behavioral science. Our findings can be used to develop behavior change strategies and policies that are responsive to target populations.

## Methods

### Study Design

Twitter is a social media platform that allows users to microblog and socially network. Each user is allowed up to 280 characters in a post (called a tweet). Users can post text, pictures, videos, or links to websites. Users who have registered for an account can tweet, like, and comment on another user’s tweet and repost tweets (called a retweet). Registered users can also follow accounts and send private messages to each other. Unregistered users can read tweets, retweets, and comments but cannot engage in any interactions [23].

Twitter was selected because of its ability to capture real-time data [19]. Other studies have used Twitter to capture data on vaccine hesitancy. One study compared survey results about vaccine hesitancy in 2018 (before the COVID-19 pandemic) to data captured from Twitter and found that the data were similar to each other [24]. The study argued that Twitter could potentially be used instead of surveys in some contexts and similar results would be obtained [24]. Another study went as far as saying that Twitter is a “sentinel tool” for identifying public opinions on vaccinations [25]. Thus, Twitter was selected as the site of data collection because it offers a publicly available repository of discourse data (ie, tweets) that are captured in a specific point in time for a specific geographic area.

This study did not require research ethics approval, as it was based on data that were publicly available. Other Canadian-based studies [26] have forgone ethical review by using publicly available Twitter data, as some sources are anonymous or unidentifiable. Only the Twitter user’s username (ie, handle), city or town, and tweet content were extracted. This paper only presents aggregated data. Moreover, no interaction occurred between the authors of this study and any of the Twitter users.

### Data Collection

After the researcher (JG) was approved for a developer account on Twitter, she received credentials for accessing Twitter’s application programming interface. By using a Jupyter environment, the researcher created a Python program to access Twitter’s application programming interface. Twitter allows access to tweets up to 1 week after they are posted. Thus, the researcher collected data from two time periods (December 18 and 23, 2020) to access 2 weeks’ worth of tweets. Tweets that contained the words “COVID” and “vaccine” were extracted. Similar to a library search, tweets were returned based on variations of these words, such as “COVID-19,” “COVID19,” “vaccination,” and “vaccinate.”

**Figure 1:**
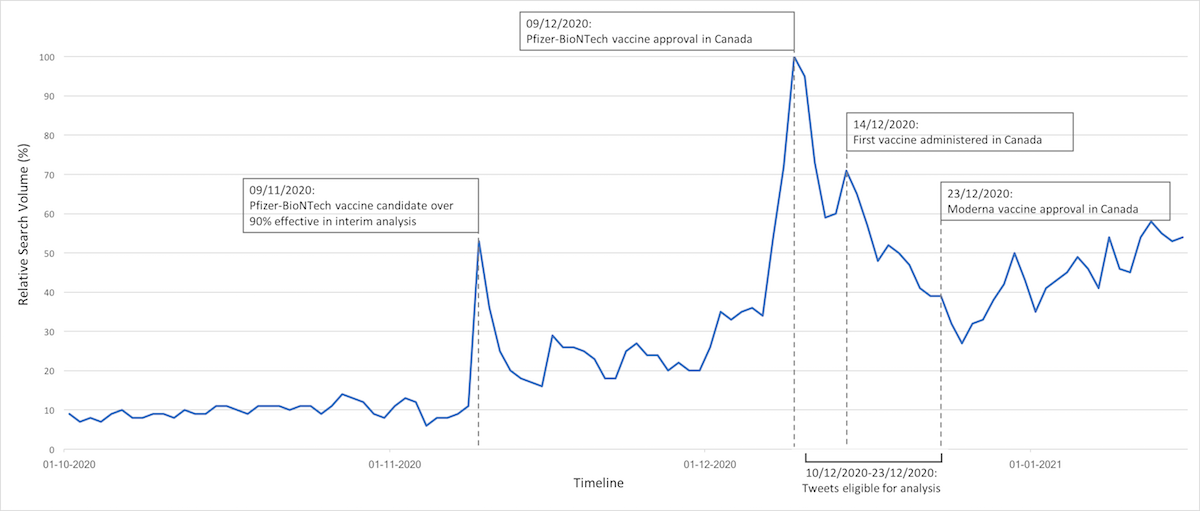
A graph depicting Google Trends data for the combined search terms “covid” and “vaccine” aligning with vaccine approval and administration dates in Canada. Tweets that were posted between December 10 and 23, 2020 were eligible for analysis. This date range aligned with the time when the highest peaks in related Google search activity occurred in Canada. This figure indicates that the number of searches on Google for the combined words “COVID” and “vaccine” was highest in December 9, 2020. All other searches were relative to this highest peak. For example, on December 14, 2020, roughly 70% of related searches occurred in December 9, 2020 [28]. It was not possible to obtain more detailed numbers.

Data were extracted from tweets from December 10, 2020, to December 23, 2020. These dates were selected because they followed the Pfizer-BioNTech vaccine approval announcement in Canada (December 9, 2020) and included the dates for the first vaccine administration in Canada (December 14, 2020) and the approval of the Moderna vaccine in Canada (December 23, 2020). This date range also accounted for the time frame when the highest number of searches for terms that included both “COVID” and “vaccine” occurred on Google, which perhaps indicated a spike in interest [27]. Thus, our data reflects a time period when receiving a COVID-19 vaccine was close to becoming a reality. Figure 1 provides a graph that shows when data were extracted and when COVID-19–related events occurred in Canada.

**Figure 2:**
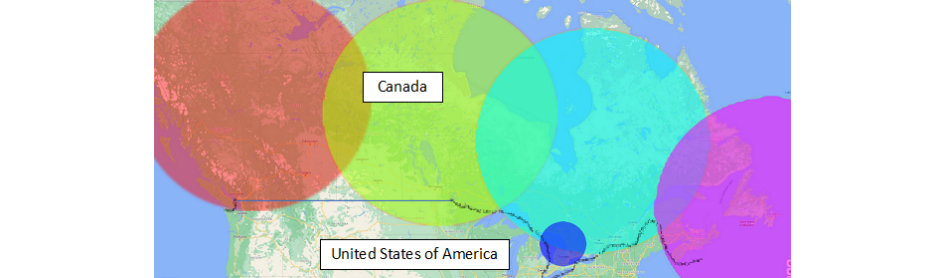
A map of where Twitter users were located. Tweets from outside of Canada (ie, those from the United States) were excluded.

To only include tweets from Canada, the researchers used five geographic radiuses that covered most of Canada. However, several small areas were unintentionally omitted (Figure 2). It was not possible to know how many tweets were missed.

Demographic data beyond users’ locations (ie, city or town) were not collected. It was possible to obtain estimates for other demographic information, such as age and gender, from third-party companies. However, this study was operating within the confines of publicly available data so as to disseminate the findings sooner.

### Data Analysis

The results were exported to a comma-separated values file and were analyzed in Microsoft Excel. Tweets were randomized (ie, reordered) in Excel so that tweets were not included based on date. As we expected, the number of tweets extracted was insurmountably high for manual analysis. Therefore, we randomly selected 20% of the tweets to be screened for eligibility. This exceeded the number of randomly selected tweets in other studies, which only included 10% of returned tweets for screening [28]. Double screening was performed for 10% of the tweets to ensure consistency. Manual analysis was selected because this study was exploratory in nature; it was unclear what themes might emerge a priori. As such, training an automated analysis program was unfeasible.

Eligible tweets included any tweets from a Canadian location that contained an expression of hesitancy toward COVID-19 vaccines. These included tweets that provided links to articles or other media that expressed hesitancy toward any COVID-19 vaccine. Eligible tweets also included those with graphics that expressed sentiments of COVID-19 vaccine hesitancy. Tweets that expressed positive or unclear sentiments toward COVID-19 vaccines were excluded. Tweets captured from the United States (given the country’s geographic proximity to Canada) were also excluded. As data were extracted on two dates, several duplicate tweets were present. These were identified and deleted in Excel.

All tweets that were deemed eligible after screening were analyzed (ie, qualitatively coded) by 2 authors (JG and HMVM). These researchers had expertise in qualitative coding. Additionally, 10% of the eligible tweets were double-coded to ensure consistency.

In Excel, a content analysis was performed on all eligible tweets. The majority of health studies that use Twitter data (56%) have conducted content analyses [18]. Content analysis was performed as described by Sutton et al [28]; the content of each tweet was systematically reviewed by at least 1 researcher. The researcher(s) then coded the content of tweets according to their meaning. The resulting codes were then organized into thematic categories. Each eligible tweet could be coded into one or more themes.

Once themes emerged from the content analysis, they were mapped onto the TDF. The TDF was selected because it applies a theory-based approach to understanding behavior and has been used extensively in implementation science research. The TDF consists of the following 14 domains: knowledge; skills; social and professional roles and identities; beliefs about capabilities; optimism; beliefs about consequences; reinforcement; intentions; goals; memory, attention, and decision processes; environmental context and resources; social influences; emotion; and behavioral regulation. It has been used in other research pertaining to seasonal flu [29] and human papillomavirus vaccine hesitancy [13] to identify barriers to vaccine uptake and plan for implementation interventions.

## Results

### Tweet Characteristics and Themes

**Figure 3:**
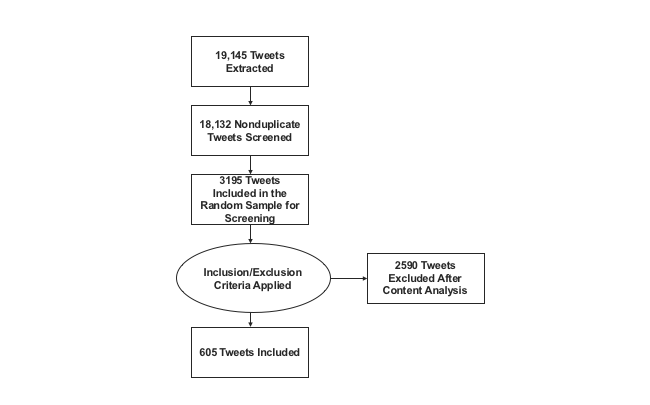
Modified PRISMA (Preferred Reporting Items for Systematic Reviews and Meta-Analyses) diagram of the data extraction process.

In total, 18,132 tweets were returned as search results. Overall, 3915 tweets were screened for eligibility. These tweets represented 21.6% of the total number of tweets. It took approximately 1 hour to manually screen 100 tweets. The 10% (400/3915) of tweets that were double-screened resulted in a Cohen κ coefficient of 0.89, indicating an almost perfect agreement. After screening, 605 tweets met the inclusion criteria. This was represented in a modified PRISMA (Preferred Reporting Items for Systematic Reviews and Meta-Analyses) diagram (Figure 3).

Through content analysis, the included tweets were grouped into the following major themes concerning vaccine hesitancy: safety, political skepticism, influence from authority figures, a lack of knowledge, and legal liability. The final theme included medical legacies. This theme was different from the other categories of vaccine hesitancy. The themes were not mutually exclusive. Examples of tweets were not provided with the presentation of the themes to preserve the anonymity of Twitter users. In the following subsections, each theme will be described.

### Safety

Overall, 48.3% (292/605) of tweets were about safety. These were largely centered around the worry that the vaccine would cause more harm than good. These tweets also expressed concerns that the COVID-19 vaccine was developed more quickly than other vaccines and that the COVID-19 vaccine was not tested to the same rigorous extent as other vaccines. Apprehension over severe side effects was also noted from tweets, including those that reported on nurses fainting and vaccine trial participants experiencing Bell palsy.

### Political Skepticism

Another major theme found in 32.4% (196/605) of tweets was skepticism toward the political motivations behind vaccine development. Several Twitter users presented conspiracy theories about the COVID-19 vaccine being a vehicle for exerting political control over citizens. Other participants felt that the vaccine was not tested enough due to political pressures to reopen the economy. Several Twitter users in Canada were also highly influenced by politics in the United States; they cited rumors about the White House threatening the leadership of the US Food and Drug Administration to rush vaccine approval or face forced resignation. Tweets also indicated concern over the influence of big, government-backed pharmaceutical companies (“Big Pharma”) that were motivated by profits instead of the desire to help people.

### Deficits in Medical and Epidemiologic Literacy Concerning the Benefits of Vaccination

Many tweets (159/605, 26.3%) indicated a lack of knowledge about vaccines among Twitter users. For example, several users expressed the idea that if those who contracted COVID-19 had a ≥99% survival rate, then they should not have to receive a vaccine that is said to be 95% effective. Additionally, Twitter users questioned why anyone else should be concerned if they do not receive the vaccine, indicating a lack of understanding of herd immunity. Twitter users also reported concerns about how the vaccine would alter human DNA. Several Twitter users also felt that a lack of a vaccine for cancer, heart disease, and AIDS was proof that a new virus could not be cured. Additionally, Twitter users viewed COVID-19 as a mild disease; therefore, their interest in undergoing vaccination was low.

### Authority Figures

Another theme we found was mistrust toward the COVID-19 vaccine resulting from Canadian and international authority figures not taking the vaccine (51/605, 8.4%). For example, several tweets highlighted users’ mistrust toward the CEO of Pfizer and political figureheads in Canadian politics like Doug Ford (the elected provincial leader of Ontario), as they were not taking the vaccine. However, later tweets criticized public figures such as Dr Bonnie Henry (the Provincial Health Officer of British Columbia) and Alexandra Ocasio-Cortez (a member of the US House of Representatives) for receiving the vaccine before frontline workers and older adults.

### Legal Liability

To a smaller extent (19/605, 3.1%), Twitter users also expressed mistrust toward vaccines that was based on reports of not being able to take legal action against drug companies if a person experiences any side effects. Additionally, news of the Federal Vaccine Injury Compensation Program in Canada resulted in further skepticism toward vaccine safety.

### Medical Legacies

The final theme was unlike all of the other themes of vaccine hesitancy in this paper—the legacy of harm caused by health care institutions that have traditionally targeted the Black, Indigenous, and people of color (BIPOC) community and the lesbian, gay, bisexual, transgender, queer+ (LGBTQ+) community. Tweets (24/605, 4%) in this theme highlighted the lack of trust toward the COVID-19 vaccine resulting from how marginalized groups, such as the BIPOC and LGBTQ+ communities, have been historically targeted by the medical community. For example, the Tuskegee syphilis experiments were referenced in several tweets. Moreover, the first people who were vaccinated in the United States were Black health care workers, and several Twitter users viewed this as forced participation in medical experiments. Additionally, a poster promoting COVID-19 vaccination was viewed as paralleling the stigmatization of people who take pre-exposure prophylaxis, a medication for people living with HIV.

### Theoretical Domains Framework

**Figure 4:**
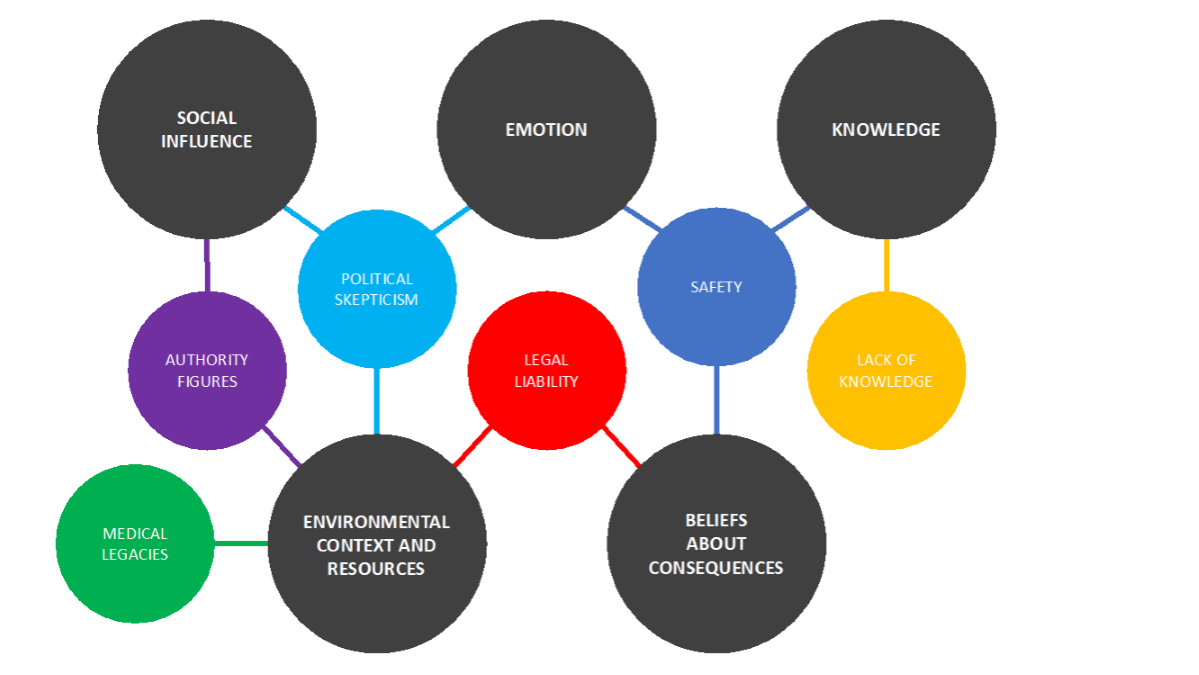
Themes were categorized based on the TDF. The TDF domains are represented by the dark-gray circles. The themes from the content analysis (smaller colored circles) were mapped to relevant TDF domains. TDF: Theoretical Domains Framework.

Themes were mapped to the TDF and categorized into the following five domains: knowledge, beliefs about consequences, environmental context and resources, social influence, and emotion. The mapping of themes to TDF domains was an interpretive and consensus-driven exercise that was conducted by two study authors (JG and HM). Disagreement was reconciled by a third study author (HMVM). Figure 4 displays a representation of the themes that were mapped to the TDF. We provide insight into this framework in the *Discussion* section. Overall, themes were not mutually exclusive; themes were classified according to several TDF domains.

## Discussion

### Principal Results

Through content analysis and TDF application, this study identified the reasons behind vaccine hesitancy among Twitter users in Canada. The major themes that emerged included concerns over safety, suspicion about political or economic forces driving the COVID-19 pandemic or vaccine development, a lack of knowledge about the COVID-19 vaccine, messages from authority figures, and a lack of legal liability from vaccine companies. An additional theme regarding the historical impact of medical mistrust among marginalized communities was also presented. These themes were categorized into the following five TDF constructs: knowledge, beliefs about consequences, environmental context and resources, social influence, and emotion. Thus, efforts to overcome vaccine hesitancy should focus on targeting these constructs.

Although evidence concerning vaccine hesitancy toward the COVID-19 vaccine is still emerging, our findings are consistent with previous studies. A study from Israel found that COVID-19 vaccine hesitancy was related to concerns about safety and efficacy and the belief that the disease is mild [5]. This was similar to our study, wherein concerns about safety was the top reason for vaccine hesitancy. The efficacy of the vaccine and the belief that the virus is mild were grouped into the lack of knowledge theme, which was another top reason for vaccine hesitancy in our study. Another study surveyed individuals from Canada and the United States in May 2020 and reported that vaccine hesitancy correlated with a lack of trust about a vaccine’s benefit, concerns about safety (ie, unknown future health consequences), commercial profiteering, and a belief in natural immunity [6]. Of note, these respondents were more likely to receive a vaccine if there was evidence of rigorous testing and safety measures [6]. Both of these studies were conducted prior to the development and implementation of a COVID-19 vaccine. As such, their results were hypothetical.

This study identified the particular reasons why people in Canada may be hesitant to receive a vaccine, so that implementation scientists who are responsible for vaccine rollouts can become responsive to these concerns. Although the analyzed tweets were from Canada, we believe that the tweets’ themes may be generalizable to other contexts. To our knowledge, no other study has analyzed tweets to determine the reasons behind COVID-19 vaccine hesitancy. This study’s contribution is especially important because the timing of our study coincided with the approval of the first two vaccines (ie, the Pfizer-BioNTech and Moderna vaccines) and the first vaccine administration in Canada.

Our results relate to vaccine hesitancy in general (ie, past research on non–COVID-19 vaccines), as prior related research has provided similar findings. For example, the influence of the media and people’s knowledge about vaccines, past experiences, perceptions of risk, and trust have all been documented [30]. However, hesitancy toward the COVID-19 vaccines presents new, unprecedented challenges; namely, the global COVID-19 pandemic is unlike any pandemic that has been experienced in the past century, herd immunity depends on vaccine participation on a global scale, and new SARS-CoV-2 strains can emerge if the virus has opportunities (ie, time and vectors) to mutate. Additionally, the long-term health consequences of COVID-19 are unknown [31].

Our recommendation for the organizations responsible for implementing vaccination programs is to create behavioral interventions that are responsive to the concerns presented in this study. The mapping of these themes to the TDF provided us with preliminary insights into how to best target these behavioral interventions. For example, safety was a top concern that was found in the tweets, and we mapped safety to both knowledge and beliefs about consequences. Thus, targeting vaccine literacy may be beneficial, and this can be done by explaining how vaccines work, why they are safe, and how no steps were missed in the expedient process of COVID-19 vaccine development. However, trust in politicians and pharmaceutical companies is a vaccine hesitancy factor that is difficult to target because both groups are necessarily involved in vaccine rollouts. One approach to targeting this concern might be to have trusted physicians speak to their patients about why it is important to be immunized. This approach falls under the domain of emotion in the TDF.

Although providing details on interventions for responding to vaccine hesitancy was beyond the scope of this study, Table 1 provides example suggestions for interventions based on each TDF domain.

More research is necessary to determine whether addressing these concerns is effective in overcoming vaccine hesitancy.

**Table 1:** Reasons for vaccine hesitation fell under several Theoretical Domains Framework (TDF) constructs (left column). The rightmost column provides examples of intervention suggestions for responding to vaccine hesitancy in relation to the TDF construct.

TDF constructs	Content analysis theme	Example suggestions
Knowledge	Lack of knowledge	Introduce campaigns that educate the public about using clear language in media that are commonly used to digest content (eg, social media).
Social influence	Authority	Have nonpolitical, respected older adult Canadian celebrities take the vaccine as an example. Such celebrities could be retired athletes or musicians.
Environmental context and resources	Political skepticism	Emphasize that vaccines are rooted in science and not politics. This is a difficult quality to understand. In action, this could be done by having messages come from trusted physicians instead of politicians.
Emotion and beliefs about consequences	Safety	Highlight examples of instances when the vaccine has worked. Reiterate the safety of the vaccine. Reiterate the fact that the steps in the scientific development of the vaccine were not missed.

### Limitations

As of January 2021, roughly 6.45 million (~17%) Canadians use Twitter [32]. Therefore, the perspectives on vaccine hesitancy presented in this paper are not wholly representative of the perspectives of all people in Canada. All users included in this study represent people in Canada with broadband internet access, which, as the COVID-19 pandemic has illustrated, is an important determinant of health [33]. As such, we likely missed the perspectives of those who face challenges when accessing the internet. It is also possible that nonhuman Twitter users (bots) were represented in our sample. Previous research has found that Twitter bots have manipulated public opinion and fueled cascades of negative emotions related to topics about COVID-19 [34]. Without any way to systematically identify and exclude these tweets, we suspect that several such tweets were included in our analysis. We also searched for English-only tweets due to limitations in language expertise among this study’s authors. A more comprehensive content analysis that is representative of all people in Canada should include tweets that are written in other languages. This limitation may have resulted in themes not being identified, including those related to culturally specific concerns.

It was not possible to collect demographic data such as age, gender, and ethnicity while also preserving users’ anonymity. Thus, we were unable to analyze the demographic characteristics of Twitter users who expressed vaccine hesitancy.

Although the search strategy could have been expanded to include many more terms related to vaccination (eg, “shot,” “jab,” “immunization,” etc), the search results would have been insurmountable for conducting our manual analysis process. Additionally, terms related to hoax beliefs were not included; the inclusion of such terms would have likely produced more results. Although saturation was achieved for our search, we may have missed themes that used alternative language to express vaccine hesitancy.

Of note, the examples of interventions presented in Table 1 are merely suggestions. A behavioral scientist may have more informed suggestions about how to combat vaccine hesitancy according to the TDF.

Finally, the tweets related to the medical legacies discussed in this paper should not be viewed as tweets about vaccine hesitancy or conflated with those under the categories of safety, a lack of knowledge, political skepticism, messages from authority figures, and legal liability. As Mosby and Sridrovich [35] have emphasized, health care providers need to understand the history of “racially segregated health care and medical experimentation.” Additionally, Boyd [36] stated that the “hyper-focus on hesitancy implicitly blames Black communities for their undervaccination, and it obscures opportunities to address the primary barrier to COVID-19 vaccination: access.” Building trust in the medical system goes far beyond the suggestions presented in this paper.

### Conclusions

Overall, this study identified the reasons why people in Canada may feel hesitant toward receiving a COVID-19 vaccine. These reasons fell under the following themes: safety concerns, suspicions about political or economic forces, a lack of knowledge, messages from authority figures, and a lack of legal liability from vaccine companies. Additionally, other tweets revealed the historical impact of medical mistrust among marginalized communities, which should not be viewed as hesitancy or as the result of the reasons identified in this paper. Overall, behavioral, implementation, and public health scientists can use theory-based approaches like the TDF to design interventions that are tailored to address the concerns that people have and improve the uptake of the COVID-19 vaccine, thereby increasing the chances of achieving the threshold necessary for herd immunity.

## Abbreviations

BIPOC: Black, Indigenous, and people of color
LGBTQ+: lesbian, gay, bisexual, transgender, queer+
PRISMA: Preferred Reporting Items for Systematic Reviews and Meta-Analyses
TDF: Theoretical Domains Framework

## References

[1] Detoc M, Bruel S, Frappe P, Tardy B, Botelho-Nevers E, Gagneux-Brunon A (2020). Intention to participate in a COVID-19 vaccine clinical trial and to get vaccinated against COVID-19 in France during the pandemic. Vaccine.

[2] Harrison EA, Wu JW (2020). Vaccine confidence in the time of COVID-19. Eur J Epidemiol.

[3] Ten threats to global health in 2019. World Health Organization.

[4] Angus RI (2020). More Canadians willing to roll up their sleeves right away as national COVID-19 vaccine rollout begins. Angus Reid Institute.

[5] Dror AA, Eisenbach N, Taiber S, Morozov NG, Mizrachi M, Zigron A, Srouji S, Sela E (2020). Vaccine hesitancy: the next challenge in the fight against COVID-19. Eur J Epidemiol.

[6] Taylor S, Landry CA, Paluszek MM, Groenewoud R, Rachor GS, Asmundson GJG (2020). A proactive approach for managing COVID-19: The importance of understanding the motivational roots of vaccination hesitancy for SARS-CoV2. Front Psychol.

[7] (2020). Coronavirus disease (COVID-19): Herd immunity, lockdowns and COVID-19. World Health Organization.

[8] Majority of Canadians intend to get the COVID-19 vaccine, September 2020. Statistics Canada.

[9] Tilburt JC, Mueller PS, Ottenberg AL, Poland GA, Koenig BA (2008). Facing the challenges of influenza in healthcare settings: the ethical rationale for mandatory seasonal influenza vaccination and its implications for future pandemics. Vaccine.

[10] Gostin LO, Salmon DA, Larson HJ (2021). Mandating COVID-19 vaccines. JAMA.

[11] Glanz K, Bishop DB (2010). The role of behavioral science theory in development and implementation of public health interventions. Annu Rev Public Health.

[12] Atkins L, Francis J, Islam R, O'Connor D, Patey A, Ivers N, Foy R, Duncan EM, Colquhoun H, Grimshaw JM, Lawton R, Michie S (2017). A guide to using the Theoretical Domains Framework of behaviour change to investigate implementation problems. Implement Sci.

[13] Garbutt JM, Dodd S, Walling E, Lee AA, Kulka K, Lobb R (2018). Theory-based development of an implementation intervention to increase HPV vaccination in pediatric primary care practices. Implement Sci.

[14] Jackson C (1997). Behavioral science theory and principles for practice in health education. Health Educ Res.

[15] Bandura A (1977). Social Learning Theory.

[16] Denecke K, Krieck M, Otrusina L, Smrz P, Dolog P, Nejdl W, Velasco E (2013). How to exploit twitter for public health monitoring?. Methods Inf Med.

[17] Parker J, Wei Y, Yates A, Frieder O, Goharian N (2013). A framework for detecting public health trends with Twitter. Proceedings of the 2013 IEEE/ACM International Conference on Advances in Social Networks Analysis and Mining.

[18] Sinnenberg L, Buttenheim AM, Padrez K, Mancheno C, Ungar L, Merchant RM (2017). Twitter as a tool for health research: A systematic review. Am J Public Health.

[19] Scanfeld D, Scanfeld V, Larson EL (2010). Dissemination of health information through social networks: twitter and antibiotics. Am J Infect Control.

[20] Leading countries based on number of Twitter users as of October 2020. Statista.

[21] Roth Y, Pickles N (2020). Updating our approach to misleading information. Twitter.

[22] Lunden I (2020). Twitter prioritizes blue-check verifications to confirm experts on COVID-19 and the novel coronavirus. TechCrunch.

[23] New user FAQ. Twitter.

[24] Nowak SA, Chen C, Parker AM, Gidengil CA, Matthews LJ (2020). Comparing covariation among vaccine hesitancy and broader beliefs within Twitter and survey data. PLoS One.

[25] Tavoschi L, Quattrone F, D'Andrea E, Ducange P, Vabanesi M, Marcelloni F, Lopalco PL (2020). Twitter as a sentinel tool to monitor public opinion on vaccination: an opinion mining analysis from September 2016 to August 2017 in Italy. Hum Vaccin Immunother.

[26] Al-Rawi A, Siddiqi M, Morgan R, Vandan N, Smith J, Wenham C (2020). COVID-19 and the gendered use of emojis on Twitter: Infodemiology study. J Med Internet Res.

[27] covid vaccine. Google Trends.

[28] Sutton J, Vos SC, Olson MK, Woods C, Cohen E, Gibson CB, Phillips NE, Studts JL, Eberth JM, Butts CT (2018). Lung cancer messages on Twitter: Content analysis and evaluation. J Am Coll Radiol.

[29] Kenny E, McNamara Á, Noone C, Byrne M (2020). Barriers to seasonal influenza vaccine uptake among health care workers in long-term care facilities: A cross-sectional analysis. Br J Health Psychol.

[30] Dubé E, Laberge C, Guay M, Bramadat P, Roy R, Bettinger J (2013). Vaccine hesitancy: an overview. Hum Vaccin Immunother.

[31] Long-term effects of COVID-19. Centers for Disease Control and Prevention.

[32] Distribution of Twitter users in Canada as of January 2021, by gender. Statista.

[33] Benda NC, Veinot TC, Sieck CJ, Ancker JS (2020). Broadband Internet Access Is a Social Determinant of Health!. Am J Public Health.

[34] Shi W, Liu D, Yang J, Zhang J, Wen S, Su J (2020). Social bots' sentiment engagement in health emergencies: A topic-based analysis of the COVID-19 pandemic discussions on Twitter. Int J Environ Res Public Health.

[35] Mosby I, Swidrovich J (2021). Medical experimentation and the roots of COVID-19 vaccine hesitancy among Indigenous Peoples in Canada. CMAJ.

[36] Boyd R (2021). Black people need better vaccine access, not better vaccine attitudes. The New York Times.

